# Perceptions from pharmaceutical stakeholders on how the pharmaceutical budget is allocated in South Africa

**DOI:** 10.1186/s40545-021-00362-3

**Published:** 2021-09-21

**Authors:** Lirosha Moodley, Fatima Suleman, Velisha Ann Perumal-Pillay

**Affiliations:** Discipline of Pharmaceutical Sciences, College of Health Sciences, University of KwaZulu-Natal, Private Bag X54001, Durban, 4000 South Africa

**Keywords:** Healthcare budget, Burden of disease, Pharmaceutical expenditure, Standard treatment guidelines, Essential medicines list, South Africa

## Abstract

**Background:**

South Africa faces a heavy burden of disease, which impacts resource allocation. The needs of South Africa require efficient translation into pharmaceutical expenditure for medicine provision, to ensure availability of medicines. Given that South Africa faces various challenges with medicine provision accompanied by rising pharmaceutical expenditure, this study aimed to report on the considerations and methods used to determine the healthcare budget for South Africa, and how it is translated into pharmaceutical expenditure for medicines provision on the Standard Treatment Guidelines and Essential Medicines List and non-essential medicines in the public sector.

**Method:**

Qualitative, semi-structured interviews guided by a discussion guide were conducted with seven pharmaceutical officials involved in the budget and resource allocation process, between October 2019 and March 2020. Interviews were recorded and transcribed verbatim. Once the interviews were coded by the first author they were verified by the other authors. Data were thematically analysed.

**Results:**

This study depicted the knowledge and participation of pharmaceutical services in the budget process. The National and Provincial Department of Health have improved pharmaceutical budgeting by making strides towards a collaborative, informed, and more evidence-based approach. Pharmaceutical services have roles in advising on requirements; commenting where necessary, constantly monitor and taking accountability for their budget. The main considerations that determined the budget included population size and growth, historical expenditure, the extra heavy burden of disease and incidence rate, demand data and forecasting. The local and provincial pharmacy and therapeutics committee play a vital role in monitoring the budget and expenditure; ensuring adherence to guidelines; controlling the extent to which non-Essential Medicine List items are used and advising accordingly.

**Conclusion:**

This was the first study to report on the decision and thought processes of the healthcare budget and its translation into pharmaceutical expenditure for medicine provision in South Africa. Many factors were considered to inform the budget, with the Standard Treatment Guideline and Essential Medicines List being the principal guide for medicine provision. This process was well-controlled and monitored by the pharmaceutical therapeutics committee. Documenting the South African experience can assist other countries in their budget decisions for medicines.

## Background

The healthcare budget is a plan of allocating resources to produce the best possible outcome given the revenue. A health budget is a complex tool that includes key financial objectives and commits to implementing health policies and strategies. The budget is developed by the Minister of Finance and related members. The Minister of Health plays a crucial role in preparing priority orientated budget proposals [1].

A good health budget is one where health priorities and the allocation of resources are aligned. South Africa (SA) faces a quadruple burden of disease being: communicable diseases such as human immunodeficiency virus (HIV), acquired immunodeficiency syndrome (AIDS) and tuberculosis (TB); maternal and child mortality; non-communicable diseases (NCD) such as hypertension, diabetes, cancer, mental illness, chronic lung conditions and trauma and injury [2]. The healthcare budget needs to be aligned with these conditions, to ensure the healthcare needs of the population are met. Other considerations when formulating the budget should include, analysis of expenditure reports against expected revenues to determine if there could be possible increases in planned expenditure. A clear and realistic budget proposal can then be drafted [1].

The World Health Organization (WHO) developed a handbook on strategizing national health in the twenty-first century. Chapter 8 of this handbook focussed on budgeting for health. It provided an in-depth look into guiding principles, budget planning and enabling factors to ensure alignment of the budget and health priorities and resources. This handbook focuses on the public sector, serves as an essential guide for health planners and managers and is in accordance with progress towards universal health coverage (UHC) [1].

**Fig. 1:**
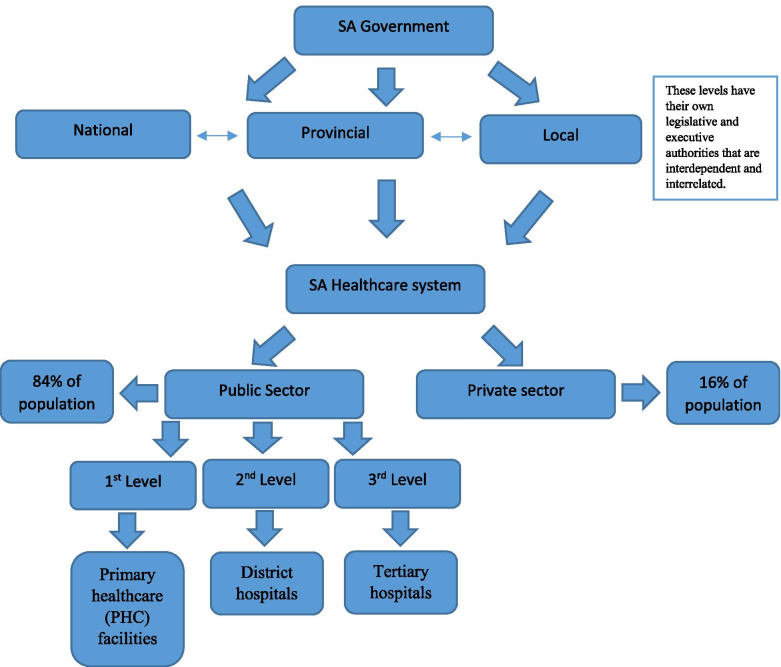
South African government and healthcare system [8–11]

When considering the healthcare budget in the context of SA, the structure, financial and economic status of the country needs to be viewed. SA is a democratic country with a 3-tier system of government (Fig. 1).

The National and Provincial Government of SA use the Public Finance Management Act (PFMA) to regulate finances. This act ensures revenue, expenditure and liabilities are managed efficiently [3]. SA follows the Division of Revenue Act (DORA), the purpose of the DORA is to provide an equitable division of revenue raised nationally, among National, Provincial, and local government entities. This is done on an annual basis. The Act is required to determine from the National Revenue, the National, Provincial and municipal shares and any other allocations and conditions they come with [4].

Private and public pharmaceutical expenditure also have disparities. The pharmaceutical sector faced various challenges including, inequity in access to essential medicines, rising drug prices and irrational medicine use to name a few [5]. In an attempt to remedy these challenges amongst others the SA government developed the National Drug Policy (NDP). The goal of the NDP was to provide a plan to ensure an 'adequate and reliable supply of safe, cost-effective drugs of acceptable quality and the rational use of drugs by prescribers, dispensers and consumers’ [5]. This policy recommended the development of pricing policies/interventions for medicines used in SA; development of the Standard treatment guidelines (STG) and an essential medicines list (EML) as a guide to ensure rational medicine use and reduce over-expenditure on pharmaceuticals; the use of generic medicines and effective procurement and distribution of medicines to name a few [5].

To promote the rational choice of drugs in SA the NDP called for the creation of the National Essential Medicine List Committee (NEMLC) which was appointed by the Minister of Health. This committee was responsible for the selection of medicines in the public sector [6]. The selection of medicines is based on the health needs of the majority of the population, effectiveness, safety and risk–benefit ratio of the medicine [5]. A healthcare budget is meant to ensure the availability of essential medicines in the public sector.

The aim of this study, therefore, was to determine how the healthcare budget is calculated for the population of SA and translated into pharmaceutical expenditure for medicines provision on the STG/EML and non-EML items. Achieving this aim will allow documentation of the budgeting process; discover deficiencies and shortfalls, if any, and determine if there is consistency throughout the country. This will ultimately achieve the main aim of this research: to strengthen health systems and promote continued progress in health financing.

## Methods

### Sample selection

This study focused on healthcare budgeting and its translation into pharmaceutical expenditure in a South African setting. This required interviewing pharmaceutical services representatives from all nine provinces in SA who had intimate knowledge of the budgeting process and decision-making. Invitations to participate in this study were sent to 10 individuals, who are the key individuals for their provinces as representatives in that committee, of which only 8 responded, 7 agreed to participate, and 1 withdrew from the study. This represented seven of the nine provinces. Semi-structured interviews were conducted with 7 participants.

### Development of the instrument

The interviews were guided by a discussion guide developed by the researchers. The first draft of the discussion guide was piloted with the first participant interviewed, who had the longest experience in the position. The discussion guide was then amended to ensure questions were clear and concise. The guide included semi-structured open-ended questions regarding members involved in the budget process; participants roles and responsibilities; knowledge on the calculation of the healthcare budget for the burden of disease; and its translation into pharmaceutical expenditure for provision on the STG/EML and non-EML items and knowledge on any analysis done with regard to the health budget and the STG/EML. Based on the questions asked below knowledge and perception were assessed and conclusions were derived.

**Box 1:** Discussion guide for interviews with pharmaceutical officials

1. Whom are the different members involved in the budget process and how are they selected?
2. Please define your role and responsibilities as a member in the budget process
3. How is the healthcare budget, for the population, calculated for the burden of disease? Please explain the process in detail
4. How is the healthcare budget translated into pharmaceutical expenditure for provision on the STGs/EMLs as well as for non-STGs/EMLs items? Please explain the process in detail
5. How is a budget impact analysis of the cost implications of having STGs/EMLs in SA calculated? Explain in detail
6. What other health economic and/or pharmacoeconomic analyses are done, e.g. cost-effectiveness analysis for the healthcare budget?
7. What other health economic and/or pharmacoeconomic analyses are done to estimate the impacts of EML decisions?
8. What other guidelines/considerations are taken into account during these processes?
9. Anything you would like to add/say/comment on?

### Data collection

This study consisted of two arms: (a) interviews with participants on healthcare budgeting and (b) comparative analysis with WHO guidelines and SA acts and policy documents.

- The interviews were conducted by the researcher between October 2019 and March 2020. The researcher had no prior knowledge of healthcare budgeting and its translation into pharmaceutical expenditure and remained impartial to the responses. This allowed the data to be approached with openness and no expectations, to allow new perspectives to emerge [7]. All interviews were audio-recorded after obtaining informed consent from the participants. Participants were ensured that responses would be aggregated and no identifying information would be disclosed in any form during reporting of the study findings.
- The second part of this study included a comparison of the themes from the interview and the WHO budgeting for health guide, PFMA and DORA. This was done to determine if there was alignment between these guides and the responses from the interviews and served as triangulation.

### Data processing and analysis

All interviews were conducted in English. The interviews were recorded and transcribed verbatim. Transcripts were verified and analysed using content analysis methodology. Using a qualitative content analysis approach, key themes were derived partly from the questions and responses from the interviews. This method allowed large volumes of text to be broken down into structured and concise results. Patterns of meaning were identified from the transcribed interviews through four steps: condensing the data into meaning units, formulating codes from this information, developing categories from the codes and expressing an underlying meaning in the form of a theme. This was undertaken while keeping the research question and objectives in focus [7]. Transcripts were coded by the first author and verified by the other authors.

### Ethics statement

The study was granted ethics approval by the University of KwaZulu-Natal’s Biomedical Research Ethics Committee (BE541/17). All participants signed informed consent forms prior to participation. Participation was voluntary and participants were informed that they could withdraw from the study at any time without consequence. Anonymity of participants was maintained.

## Results

### Description of the sample group

Seven out of the ten individuals invited, agreed to participate. This sample represented seven of the nine provinces namely: Northern Cape, Free State, Eastern Cape, KwaZulu-Natal, Mpumalanga, Limpopo and North West. The only Provinces not represented were Gauteng and the Western Cape. Participants were from pharmaceutical or financial backgrounds. The time it took to conduct each interview ranged between 23 and 52 min. Table 1 shows the demographic characteristics of the study sample.

**Table 1:** Demographic characteristics of the study sample

Characteristics of the sample	
Gender	4—male
	3—female
Average age (in years)	50
Age range (in years)	43–69
Profession	Finance background
	Pharmacists
Range of number of years in the position	1–13 years
Mean number of years in the position	5.5 years

The key themes derived from the interviews were: (1) members involved in the budget process; (2) The budget process; (3) determination of the budget; (4) efficient usage of funds; (5) translation of the healthcare budget into pharmaceutical expenditure for provision on the STG/EML and non-EML items; (6) the budget analysis, health economic and pharmacoeconomic analysis performed with regard to the STG/EML and health budget; (7) budget and system challenges encountered. Participants were involved in all areas of the budget process in their individual provinces as well as commented on the country’s budgeting process. These are elaborated on below:

### Members involved in the budget process

#### Members at the different levels in government

When asked to describe the different members of the budget process participants responded that there were a range of members involved from both finance and health departments. These members were either from a provincial, district or institutional level. They stated that these levels were interdependent and interrelated.

“The budget inputs at the institutional level were consolidated at a district level and taken up to a provincial level.”

**Fig. 2:**
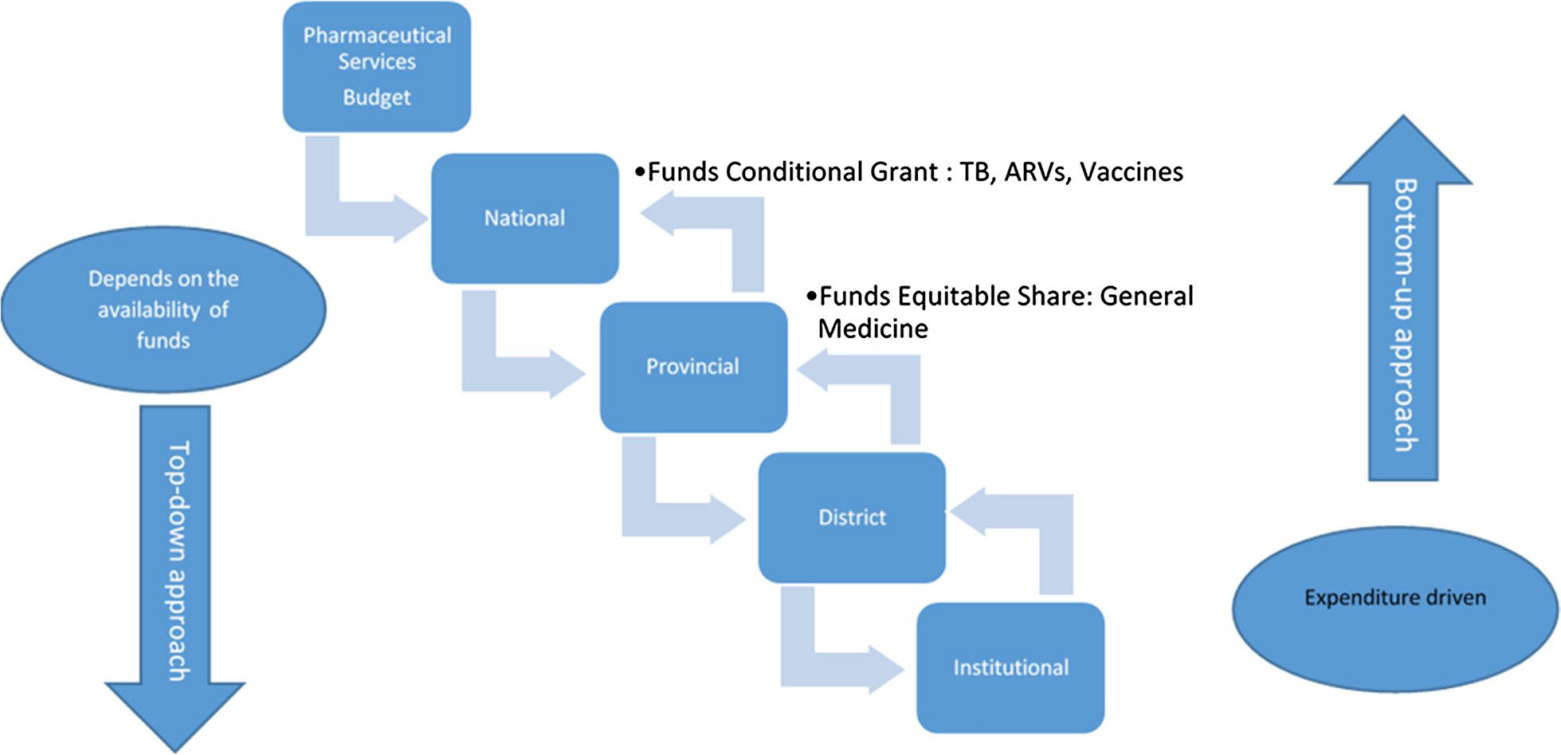
Pharmaceutical services funding and approach used by the panel

The members were involved in a “bottom-up approach which is expenditure driven or a top-down approach which depended on available funds” (Fig. 2). Members involved the budget process at the different levels in government is shown in Table 2.

**Table 2:** Members involved in the budget process at the different levels in government

Provincial	District	Institutional
Provincial treasury Chief Financial Officer (CFO) Budget committee—made up of all the chief directorates Provincial heads of pharmaceutical services Provincial medicines advisory committee—representatives from finance and supply chain Deputy director-general for health services Chief director of clinical support services Depot manager	Pharmaceutical services district manager	Pharmacy managers at an institutional level Facilities finance managers Chief executive officer (CEO) of the institution

### Roles and responsibilities of pharmaceutical services as members in the budget process

A participant explained their involvement as a part of pharmaceutical services:

“To greater or lesser degree pharmaceutical services are involved in determining the quantum required in order to cater for medicine. Sometimes it’s done unilaterally by the programme (department of finance and health) without consulting pharmaceutical services and we will just have to live with the consequences. It depends on individuals in most cases, not all departments are equally well organized to involve all potential role players in the budgeting process. If you involve yourself you can in many cases become quite an important role player in the determination of the budget.”

Another participant indicated that even though members from finance have vital roles, pharmaceutical service input is required:

“I don't think most people from budgeting, finance and supply chain understand our environment. We cannot just forecast; we also need to align our demand to our supply.”

A participant from pharmaceutical services stated that they had no involvement in the budget determination at their level:

“There isn't any budgeting process, you are just given a budget and you need to comment if the budget is sufficient or not. You don't really interrogate the budget.”

Most participants indicated that after analysis and interpretation of the budget they have three major roles namely:

1. Advisory: “Mainly advisory, because the authority of budget allocation is currently located within the budget control directorate. I give advice in terms of things that need to be factored in and triggers that you are not happy with, we need to give a motivation on why we are not happy and what would make us happy.” One participant elaborated that they are “part of the baseline budget requirement calculations, in other words using the information, enriching the information available that informs the amount of budget required. So then this was then presented to the CFO.”
2. Monitoring, a participant shared: “Monitoring needs to happen all the time, It needs to be linked to product use because rational and irrational use including non-adherence to the STG EML requires interventions in order to address and economize on available funds, whenever you lead to any decision you cannot just make an assumption on requirements without considering available funds, you are going into a complicated situation where you can only spend as much as the fiscus will allow us. The budget constantly requires analysis and constant feedback.”
3. Accountability: “I’m overall accountable for the budget that gets allocated to provide pharmaceutical services. I will be accountable for any kind of over-expenditure, under-expenditure and whatever mismanagement there may be.”

When participants were asked to describe the members involved in the budget process and how they were selected, many participants described the skills required to be a member in the budget process:

“Pharmaceutical services role players need to have the capacity to meaningfully interpret systems that are in place in order to meaningfully get input and provide input.”

Another participant described that role players involved would need to be able to:

“Look at the burden of diseases; population size; the different categories in terms of ATC (Anatomical Therapeutic Chemical) classification, and what the cost implications are, what contracts are in place, cost per unit based on your estimate from contracts, and then you will use that as an evidence-based approach to inform the budget.”

Based on the responses some participants seemed to be more involved than others. Involvement in the budget process depended on the initiative and capacity of individuals involved. Clearly defined descriptions of the financial roles of pharmaceutical services should be documented to ensure consistency between the provinces and better flow of input from pharmaceutical services. Participants responses on the budget process is reported in Table 3.

**Table 3:** Participants responses on the budget process

The budget process	
Subtheme	Comments
Budget allocations	“How the budget works in government: after the taxes are collected by SARS (South African Revenue Service), it goes to the National treasury. The National treasury will do equitable allocations between all provinces. This goes to the Provincial treasury which has its own committee of members of different departments, they allocate to different departments… Then we work from the departmental level, at department level… there will be allocations for tertiary hospital services… and then they will further break it down according to their needs.” (Fig. 3)
Provincial and National Department of Health (NDoH) allocations	“Province would get an allocation and then the Provincial treasury will do further allocations within the province. This is guided by what national says, but the allocation will be done from the Provincial treasury.” There are two different types of budgeting in the pharmaceutical budget. There is what you call the: 1. “Equitable shares—the Provincial treasury will allocate the figure, then the Provincial department of health will allocate the money according to the cost centre.” (Fig. 2) 2. “A conditional grant—will be allocated by the National Department of health. This is currently for TB/ antiretroviral (ARV) medicines and certain vaccines, we don’t buy outside the conditional grant and this is allocated to us by the National Department of health treasury.” (Fig. 2)
	When the budget is allocated to province the equitable share, conditional grant and National Tertiary Service Grant (NTSG) amounts are stipulated but the exact amounts for specific services and conditions are not indicated. The province will use their discretion from the equitable share budget on the funds required for communicable diseases, non-communicable diseases and other conditions, programmes or services. As explained by a participant: “It doesn't just appear to say it’s a portion for non-communicable diseases, it just says that this amount is for tertiary services, this amount is equitable share, and this amount is for vaccines… So those are the main score line items that they get… We use our discretion based on the previous consumption.”
Budget process timeline	All participants used a timeline for when they started planning for the financial year and when it was finalized but were not in agreement of their timelines. The allocation of funds was received by provinces at the same times, but the differences in planning could be due to the size and different requirements of the provinces
Budgeting tools/methods used	All participants stated that in the past the budget was based on historical values also known as baseline budgeting with inflation taken into account, but in 2019/20 and 2020/21 a new initiative was implemented to allow for a more evidence-based approach, this was explained in detail by participants: “You have now forecasted using the historical data but you then need to factor in what is going to happen going forward, is there going to be a reduction, or be an increase then you need to adjust for that, this is called forecast enrichment.”
	The budgeting process is being: “Revised and strengthened by accurate quantification of requirements which is based on forecasting. Processes utilizing forecasting tools and consumption-based quantification are enriched by real users such as pharmaceutical personnel, programme management and others to determine the quantity and translate it into a monetary value based on current prices.”
	“A mixed model was used. This method made use of a zero-based calculation which focused on real requirements, with demand planning being a critical function.”
	“So we work from a zero budget and we said these are the requirements.”
	A participant elaborated on the factors that have to be considered with historical budgeting: ∙ “Birth rate” (especially in the case of immunizations) ∙ “Population growth rate” ∙ “Demand data and adding adjustments” (such as inflation)
	“This data is converted into product units and then Rands (South African currency).”
	“I interact with national to give me a growth factor for a specific disease; HIV and AIDS in the district: they would give me the percentage of growth. I also get what I call the inflation medication impression, I normally take the percentage higher than the normal inflation.”
	“We usually forecast to our projections based on the actual product items that we've used for the previous two financial years, and take those quantities cash them into a rand value using the previous tender prices that we had… we'll then add the current CPI (consumer price index) to that price.”
	“Provinces will be required to provide inputs such as demand planning processes.”
	“The NDoH will look at demand data for the last three years, based on procurement and demand from facilities.”
	“Population size” and “population growth” were the greatest considerations
	“The headcount” was considered, “which includes not only patient numbers but the number of patient visits.”
	“Treasury currently uses a formula which is a population-based, to allocate the budget for the equitable share.” “The equitable share to some extent is determined by the part of the population that is not covered by medical aid. Based on the non-insured population the province will be granted a proportionate part of the total healthcare budget.”
	The level of care that you are treating a patient at will also assist the budget: “One of the factors that we need to take into account, is the nature of the level of care, meaning that at the clinic level you look at the normal types of illnesses that people have.”
Initiative to secure the pharmaceutical budget	With a more evidence-based approach to determine the appropriate budget for pharmaceutical services, a method to preserve or protect the budget can be applied. For the 2020/21 financial year, the NDoH aims to implement a ring-fenced budget for pharmaceuticals which will ensure availability of funds
	“Starting in April 2020 what is called the ring-fenced budget for pharmaceuticals will come into play.” “Ring fenced meaning protected so it’s only moved for procurement of medicine. But it will not take into account all the accruals that come from previous year.” “It gives pharmaceutical services a concrete budget to work with, at the moment that figure is fluid.” “It is something that pharmacy has been anticipating for quite a while and to date not been successful until recently where it was identified as critical to ensuring availability.” “It's going to be very crucial if you have that in a separate bank account once it's approved because by doing that, we can actually hold people accountable to that account.”
	A participant stated that even before this initiative they ring-fenced their pharmaceutical budget: “In our province, we ring-fence medicines because it is non-negotiable, we won't touch it. But the province has a right to move that money, because of other needs. We reinforce it because of limitations of resources. Only in a dire situation then the leaders will decide where to move it.”
Data sources used to determine the budget	“I was involved in the process, in terms of identifying data sources: identifying information that will enrich the numbers of the information that we get from the data analysis.” “We looked at historical consumption data and put a ± 10% Increase on previous demand data. We looked at the demand data. We went into the database, we had three sources of databases: Medicine demand or usage data was analysed using three sources: 1. RX Solutions—institutional stock management system 2. MEDSAS—depots data management system 3. LOGIS—National treasury assistant, used to procure items that are not coded, e.g. buyouts.”

**Fig. 3:**
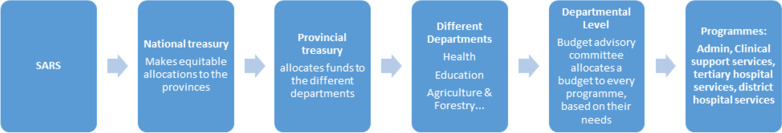
Representation of funding flows of finances in the public sector

### Considerations to inform the budget

Participants shared some of the considerations they take into account to inform budget allocation planning:

- “Alignment of demand to supply”
- “The level of care”
- “Population size”
- “Seasonal forecasting”
- “Prescriber preferences”
- “Product use”
- “Rational use of medicines—compliance with the STG/EML”
- “Extra heavy burden of disease” (ARVs/TB/NCDs)
- “Previous consumption—Historical expenditure”
- “Analysis of expired stock”
- “Compliance to STG”
- “Motivated non-EML items and progress reports”
- “Previous consumption”
- “Tender process changes, contracts, addendums or price increases.”

These considerations may require interventions to address and economize on available funds.

### Consideration of the burden of disease with regard to the healthcare budget

When discussing the burden of disease and its role in the healthcare budget, comments included:

“Some of the extra heavy burden of disease is taken into consideration. That obviously adds a huge additional burden.”

“Programmes such as TB, expanded immunization programme and HIV/AIDS programmes are considered.” “These programmes get their funding from conditional grants.”

Some participants stated they look at the burden of disease. For every disease condition they “try to take the average price of the medications available” for a specific condition at the different levels of care and “add population growth factor and inflation to it.”

Participants included that they look into conditions that are “specific to their province” and “seasonal conditions, but there are conditions they cannot predict.”

A participant felt strongly about making the burden of disease a greater consideration in the budget process:

“There is still a lot we need to look at, at national such as analysis or research or changes in the burden of disease. What’s the latest studies and how the pharmacoeconomics will affect the latest STG? The patterns in the burden of disease are not viewed. Does the burden of disease translate into cost of health in terms of the funds? Most non-communicable diseases are expensive to treat, such as hypertension, oncology treatment. There is a lack of funding, is the money being channelled according to the latest data?”. Participants responses in relation to the rational usage of funds is reported in Table 4.

**Table 4:** Participants responses on the rational usage of funds

Rational usage of funds		
	Over-expenditure of the budget	Factors affecting the budget
Comments	Some participants rarely exceeded their budget whereas others exceeded their budget often. Inconsistencies existed between participants responses in terms of accruals, some participants stated that over-expenditure would be deducted from the upcoming year’s budget. Other participants responded that they do not have accruals and senior management will assist in providing additional funds to make up for any over-expenditure	*Importance of reviewing the demand plan against the actual demand* During the interviews, a demand plan was mentioned. Participants alluded to each facility having to submit a demand plan at the beginning of the financial year to the budget office. However, they indicated that provinces were yet to review these plans against actual demand. Reviewing and monitoring this data could be beneficial to ensure good stock management; prevent over-expenditure, stock-outs and wastage
	“Exceeding the budget is not allowed. When you do this, there are legal processes that must go to a committee in parliament for approval. If they don't approve it, then it's taken from your next year's budget. But if it is condoned and there is a sound reason, treasury may look for funding to cover for that. But if it is not condoned then it will be taken from the next financial year.”	“What we had not done at the provincial level is to review the budget or the demand plan against actual demand… This will have a negative impact on their budget and also on availability because we end up procuring more money than we had estimated for. Somewhere along the way you need to cut down or need to start rationing the quantity that you procured. So if you as an institution, have additional money then you can request there is a virement shift towards non-performing programmes.”
	“The implications are that it erodes to the subsequent year’s budget. So it means you pay the previous year’s unpaid account with your new budget. We call them accruals—if expenditures or deficits are acquired over a period of time.”	“The demand planning going forward is a critical function that the provinces are embarking on, there has been capacity building by the National Department of health, through NGO (Non-governmental organization) funded consultants. Just to make sure our demand activities are strong and robust because they inform the budget.”
	Some participants stated that they rarely exceeded the budget, but in a case they foresee they do: “The HOD (head of department) actually managed to cap that, we are actually seeing things get better. The budget that we got allocated is able to push us further. Whenever we ran out of the budget they would top it up, with a little amount.”	*Rational Use of Medicines* Non-adherence to the STG and EML will have similar effects on the supplier and the facility. A participant discussed the budget impact in terms of non-adherence to the STG and EML
	Several participants shared their challenges with the budget for oncology treatment. The Oncology budget is from the equitable share and purchasing of the necessary items could consume a majority of the budget: “There is a huge challenge with oncology medication. Over the last 6 months, expenditure was overwhelming. The conditional grant is good enough for ARVs but we struggle in other areas such as oncology treatment and the unavailability of oncology items. Oncology money comes from the equitable share. I believe cancer is dominant, and we don't get treatment because the focus is still on other programmes.”	“Prescribing outside the standard treatment guideline messes up the usage. The usage is now not going to be aligned to the demand plan that was given to the supplier. The supplier will have a problem because either their product will not be used as much as it should be or it is consumed more…”
Reasoning	These inconsistencies could be due to inefficient allocations to the provinces; management prioritizing the pharmaceutical budget by making efforts to find additional funds when required; managers on the periphery informing senior management early enough to avoid a crisis and provinces managing their services according to their budget	Poor alignment of the demand plan against the actual demand as well as non-adherence to the STG/EML will have negative effects on suppliers and facilities. It may prevent suppliers from making future bids and ultimately affect availability. The pharmaceutical contracts play a vital role as they are enablers to purchasing medicines. When this process is not well managed it leads to buying off-contract, which could be costly and affect medicine availability. When contracting, the suppliers of medication are provided with an indication of the demand plan, which is informed by the STG/EML. Non-adherence to the guidelines will result in misalignment of usage and demand leading to surplus stock or stock-outs, which could have negative impacts on patients

### Translation of the healthcare budget into pharmaceutical expenditure for provision on the STG and EML and non-EML items

All participants indicated that the National STG and EML is the main guiding principal when it comes to pharmaceutical expenditure and the pharmacy and therapeutics committee (PTC) facilitate this.

“The budget is informed by the STG and EML which assist us with managing our budget, which would lead to better expenditure.”

#### Formulary list development

A participant commented:

“The NDP suggests that each province should have their own formulary list created using the National EML, which guides the prescription and consumption of medicines.”

“At a provincial level, the PTC comprises different specialities of people who amongst other activities develop a formulary list for your province based on the STG/EML. The provincial formulary consists of EML and non-EML items.”

One participant further included that once the provincial formulary has been created:

“Out of that specific list, the district needs to take from the provincial formulary, what will be their own district facility formulary and then facility.”

Another participant described how they decide on items to be included on their provincial formulary:

“The National EML and STG will have three different product analogues that are on contract. But when we are reviewing our provincial formulary, we will look at which one would be the cheapest.”

#### Role of the PTC in ensuring the STG/EML is aligned to pharmaceutical expenditure

Participants clearly defined the roles of the provincial PTC (PPTC) in ensuring the medicine budget is translated into pharmaceutical expenditure for provision on the STG/EML and non-EML items:

“The PPTC review both the budget, expenditure as well as the budget spend on what is spent.”

“The pharmaceutical expenditure on EML versus non-EML items is monitored on a quarterly basis… The PTC ensures compliance with the STG/EML to prevent misuse and over-expenditure of medication.”

“The PPTC play a major role in overseeing to what extent non-EML items should be used in the province, and the extent to which there are cost implications. That happens in both the provincial and hospital level, especially in the bigger academic hospitals, where the PTC actually review expenditure, cost, and potentially may put restrictions on the amount available for a specific programme, for specific non-EML use.”

According to another participant the PTC takes into consideration:

“The cost of the item, what is already on the formulary, and whether there are any additional benefits if we use the new molecule, and also look at the number of patients to be treated. This is an ethical debate of money versus life of and individual... Meaning when you budget in the beginning of the financial year, you budget based on your particular formulary. Sometimes the PTC will give a conditional approval…”

All participants agreed that the use of non-EML items is discouraged, but there were circumstances where they were required. Sometimes the formulary list needs to be wider than the EML due to the specialized services offered. Members on the PPTC who also sit on the National EML, take motivations discussed at the provincial level, to the national for approval. These items are sent to the National EML for review. If a decision is made at national to not approve an item it may be removed from code.

“At a national level, there is a monitoring of use on non-standard items in order to determine and raise the issue of NEMLC… in order to make a calling if such use is justified, should be covered and if there is no real value added by going outside of the EML… may make an informed decision and maybe a bit of an evidence-based recommendation that this item should not be used within the public sector.”

“Putting this product on the essential medicines list means it must be available throughout the country all the time… And there are cost implications, it must have all the considerations attended to.”

#### Use of budget for non-essential items

When participants were asked to elaborate on the provision of funds for non-EML items they discussed motivational items:

“When an item is not on the EML it is unlikely to be on contract. Then it means we must get these on quotation, this has a huge impact on the budget… They are expensive and generics are often not available.”

Clinicians are required to provide evidence to state why they require an item. This motivation will go through a bottom-up approach from the institutional PTC, to the district PTC and then the PPTC, where the entire committee will make a decision.

“It’s upon the clinician to present compelling evidence… You can procure a non-EML item for 6 months… the clinician will have to submit a progress report… if the treatment is working then we continue for another 6 months, but if the progress report is not indicating much progress, then we will no longer proceed or continue with the procurement… It is discussed at the institutional PTC, and once it is approved then depending on the funds or the availability of funds, it is escalated to the provincial PTC. At the depot, they will not place an order without the authorization from the chairperson or the secretarial PTC….”

#### Control of non-EML items

“Our approach is that we don’t spend more than 10% on non-EDLs (EMLs).”

With 1 participant stating they don't “exceed 5% by volume of items, so that's the target that we work towards, so we are given leeway, to allow all us to have 5% non-EML, so what you'll find is that non-EML items will vary in percentage-wise between provinces. The National department tells us this”. Table 5 indicates participants responses on the budget, health and pharmacoeconomic analysis. The various analyses performed by the PTC is captured in Table 6.

**Table 5:** Participants responses on the budget analysis, health economic and pharmacoeconomic analysis performed with regard to the STG/EML and health budget

The budget analysis, health economic and pharmacoeconomic analysis performed with regard to the STG/EML and health budget		
Calculation of the budget impact analysis of the actual cost implications of having an STG/EML in SA	Pharmacoeconomic analysis done for the inclusion of an item on the formulary list	The health economic or pharmacoeconomic analyses done to estimate the impacts of EML decisions
“I am not aware of this exercise being done, it may be done at national” was the general consensus by all participants	“Cost-effective analysis, cost–benefit analysis, cost-minimization analysis. Those are taken into consideration, more especially when looking at the non-EML.”	“That is only done at national. The National department is the one that does that through NEMLC.”
	“This is done on an item basis and not on the entire budget.”	At a provincial level they: “Evaluate outcomes on an input level. We only look at what will be the cost implications, for procuring and making the medicine available.”
	“If a member wants to add an item onto the formulary list, the motivation for this item must be submitted with a pharmacoeconomic evaluation” (cost–benefit, effective and minimization analysis)	Some participants stated that they do a cost analysis: “If the drug is a high cost, we will go to the PTC and try and work on the level of access. If it is expensive we must limit the access—control the level care.”
	“We will consider if it reduces the cost of treatment or the cost is the same but there is a better outcome in terms of numbers needed to treat… or the safety profile or the new drug that you are proposing to add to the formulary is better as compared to the previous one that we had.”	
	“Before you look at pharmacoeconomic evaluation clinical information is the first consideration. The indication, the safety profile of the product, if it is a replacement or addition.”

**Table 6:** Analysis performed by the PTC

Analysis performed by the PTC			
ABC analysis	ATC classification	Medicine utilization review (MUR)	Antimicrobial stewardship
The ABC analysis 'identifies a drug suspect, it might be because this item is a high expenditure or high usage item.’	“ATC classification is an international classification for medicines, based on their groupings an example would be hypertensive medications.”	“We do have an RMU sub-committee—Rational medicine utilization sub-committee, they will do the rationalization review and afterwards they will come back with interventions… they will make recommendations and implement their strategies or to reverse or fix whatever the problem is.”	“Antimicrobial resistance framework strategy is one guideline that we are trying to implement…because antibiotics are overprescribed, and we encourage facilities to use various systems before the issuing of antibiotics. So those things are being looked at to influence the budget, as well as influence the resistance that might happen.”
“We do the ABC analysis at provincial level. We look at what the depot is getting from the supplier and what the depot is issuing to the facility. At facility level, they do their own ABC analysis on what they have issued at their facility.”			
“In most cases, the facilities that have a strong PTC and are able to justify their particular usage… but these are the cost containment measures that we have to make sure that it is not misused or over-utilized. Once you have done that quarterly, we go back to our forecast…and then we will decide on all those reasons that we have picked up. You could actually go back to the facility level and counsel and improve issuing of the item to decrease expenditure.”			

### Budget and system challenges encountered

When discussing the healthcare budget for pharmaceuticals participants discussed the various challenges or shortfalls they encountered. All participants shared concerns of the allocated budget being insufficient to cater to the needs of the province. Participants found that receiving accurate data is still a challenge, systems are quite manual, and reliant on managers to provide accurate data. Electronic systems need to be more robust to access medication data in the province at any given point. The data used to determine the budget is not always reliable as expressed by some participants. The non-insured population is used to determine the budget and this is not always accurate as it fluctuates towards the end of the year when medical aid funds are exhausted, and more patients are using government services. Participants had challenges in performing analysis. The analysis is usually done by the PTC, these members have full-time demanding roles. Members may also not have the experience and skillsets to perform analysis that requires the expertise of health economists (Table 7).

**Table 7:** Participants opinions on the budgeting and system challenges encountered

Challenges	Comment
Budget allocations and its impact	“The needs expressed are far bigger than the budget you get allocated.” “We make projections based on the items that we used in the previous year. But then that does not guarantee that we’ll get that money.” “You can appeal… If you don’t get the funds you may have to make some very sad decisions.” “We may need to then make cuts on the requirements or submit to the facility to indicate that in order to stay within your allocated budget which items we need to drop, or which services we need to stop.” “Planning services around ethics criteria or access. An example would be your maximum number of patients that can be treated on a particular programme.” “You upfront decide the entry criteria for entering the specific programme or put a limitation on how much you can do.” Patients may have to be “referred to a higher level of care.” “Shift funds between programmes just to make up for the shortfalls.” (Virement shift)
System challenges	“I can talk about problem-solving and the attitude, in general, is when things go wrong that's the only time people start acting and rectifying it. When being informed about the requirements in most cases, the finance officers know better. Management will not be able to find the funds or the appropriate budgeting.”
Receiving accurate data	“It is also quite important to know, our systems are quite manual. There's other peripheral facilities, so you pretty much depend on having good managers at those peripheries. If you don't have good managers, getting data that you require might be a challenge.”
Considerations that should be viewed when determining the budget	“When we start in the third quarter…September to December, you will see a spike in the number of prescriptions, items, and medicine expenditure. And in the last financial quarter which is January to March, you will see the drop… this is an indication that we have problems with the medical aid patients… their funds are exhausted and they come to the state to continue with treatment. It is their constitutional right…We have to give them treatment. We do not budget and consider that particular trend when we are preparing our budget… it contributes to an increase in the non-EML item… you try and continue with the product they have started which are quite pricey… As a pharmacist, you have to bare the ethical burden of continuing with the treatment.”
Capacity and resources to perform further analysis	“The people who do these reviews have full-time jobs elsewhere, they just happen to be members of the committee, so their work also depends on resource availability. They participate in other activities…it depends on people being available. It's often difficult to get a hold of them.” “Certain analysis requires a health economists and is at a level a bit higher than my operational one.” “Because of limited resources, we use cost-minimization, cost–benefit, cost-effectiveness analysis. We have limited resources for that, we mainly try to use a group of clinical pharmacists that we have in the province, to do the analyses.” “Sometimes because of the long-distance, we do it telephonically.”

Participants concurred regarding their knowledge on the healthcare budget process; how the budget is calculated; allocation to pharmaceutical expenditure for the provision of medicines on the STG/EML and non-EML items; knowledge of budget, health and pharmacoeconomic analysis done and guidelines, however, a few inconsistencies in responses were identified:

- Responses differed with regard to the roles and responsibilities of the members involved, with some members having a greater involvement than others. The involvement in the budget process depended on the initiative and capacity of individuals to involve themselves in the budget process.
- Participants all worked with different timelines in terms of their budgeting process, this could be due to the size and different requirements of each province.
- Some participants stated that they rarely exceeded the budget whereas others exceeded the budget often. This could be due to insufficient budgetary allocations, poor data management on factors used to allocate the budget, accruals or poor communication of management to the relevant members.
- Over-expenditure of the allocated budget in some provinces resulted in accruals that would be deducted from the subsequent year’s budget. Other provinces were assisted with additional funds by their CFO and HOD if required. Provinces who were given additional funds may have informed their CFO and HOD early enough for them to find additional funds. The provinces who’s over-expenditure would run into accruals could be due to the province having no additional funds to cater to the over-expenditure or poor channels of communication to the relevant members. This reflects the inequities that still exist between provinces with funding allocations.

The second arm of this study consisted of a comparison of results from the interviews and the WHO budgeting for health, PFMA, and the DORA. This was done to determine if there was alignment between these guides, acts and the responses from the interviews. This also served as triangulation.

1. WHO budgeting for health

The WHO developed a guide on the importance of understanding and implementing good health budgeting practices to ensure alignment to health priorities and resources [1] (Table 8).

**Table 8:** Comparison with the WHO budgeting for health guide [1] and responses from the interviews

Budgeting for healthcare guide recommendations as per WHO	Responses from interviews
Three cycles in the budgeting process	
1. Implementation of the current year’s budget	✓
2. Planning the upcoming year’s budget	✓
3. Audit and review of the previous year	✓
Members involved in the budget process	
1. Ministries of budget/finance	✓
2. Ministries of health	✓
Roles and responsibilities	
Ministries of budget/finance: leading members for budget development	✓
Ministries of health: analysis of expenditure forecasts, preparation, presentation and negotiation of priority based budget proposals	✓
Fund flows	
Treasury ⇨ Minister of Health ⇨ Sub recipients (districts and health providers)	✓ National treasury ⇨ Provincial treasury ⇨ Department of health ⇨ Districts
Budget cycle referred to as a fiscal year which is 12 months	✓ 1 April—31 March
Budget formulation considerations	
1. Decisions consistent with macroeconomic objectives	✓
2. Aligned to health priorities	✓
3. Historical budgeting unless there are changes in the economic situation or government priorities	✓
4. Economic growth	✓
5. Inflation	✓
6. Demography	✓
7. Revenue	✓
8. Fiscal goals	✓
9. Aligned with the constitution and public financial management	✓ PFMA
Promotes performance-based budgeting	X
Access to quality budget and financial data	X This is what all provinces are aiming towards, but is still a work in progress
Medium-term expenditure framework—3-year period spending plan	✓

Based on the responses from participants and a comparison of the WHO budgeting for healthcare, SA is aligned to most of the recommendations. The two areas that still require implementations to meet the WHO's recommendations are:

i. Performance-based budgeting is promoted by WHO and based on the responses SA still seems to be using line-item budgeting. SA does try to ensure funds flow to priority areas, however performance-based budgeting requires planning, budgeting and evaluation to see measurable results. A review of the performance would assist in better allocation and spending of funds [1]. The analysis of expenditure reports against expected revenues and reviewing the demand plan against the actual demand is not done. Participants are, however, aware of this analysis and are hoping to implement it.
ii. Access to quality data: this is currently dependent on good managers on the periphery.

2. PFMA and treasure regulations

All responses were aligned to the PFMA and Treasury regulations. The participants all had a good understanding and knowledge of the financial prescripts in SA and the health budgeting process.

3. DORA

A comparison of the DORA against the responses from the interviews revealed that participants had a good understanding of the DORA and discussed the equitable share and conditional grants they received in accordance with the act.

## Discussion

### Summary of findings

A range of members from provincial to institutional, with either financial or health backgrounds or both, were interdependent and reliant on each other to inform the budget. The main roles of pharmaceutical services included: advising on requirements; commenting where necessary, monitoring and accountability for the budget allocated to them. The data observed for budgeting included: population size and growth, historical expenditure, the extra heavy burden of disease and incidence rate, demand data, forecasting and the level of care that you are treating a patient at. The budget is then allocated depending on available funds. The STG and EML is the principal guide for translation of the health budget into pharmaceutical expenditure. The PTC plays a vital role in monitoring the budget and expenditure; ensuring adherence to guidelines and controlling access to medication and the extent to which non-EML items are used.

### Comparison with other studies

This is the first study in SA to report on the determination of the healthcare budget and its translation into pharmaceutical expenditure for medicine provision. Many SA studies focussed on healthcare financing and its impact on health outcomes. With regard to the pharmaceutical expenditure in SA, studies included research on pharmaceutical pricing, purchasing policies, cost containment strategies, but not specifically how the pharmaceutical budget is allocated.

A Uganda study focussing on predictors of primary healthcare expenditure on pharmaceuticals to guide the budget process was comparable with this study. Uganda like SA makes use of an essential medicines list. The study was based in a public health setting which was in line with this SA study. The Uganda study identified predictors of pharmaceutical expenditure and their influence on estimating pharmaceutical expenditure based on the country’s needs, using pharmaceutical procurement data as opposed to interviews. The study used previous expenditure to guide the process with the necessary added adjustments based on the factors discovered. The following variables were considered when allocating the budget included: ‘OPD capita attendance, percentage of rural population below the poverty line, male literacy rate, whether a district is characterized as difficult to reach or not and the human poverty index’ [12]. In this SA study headcount of patient attendance and the uninsured population were considered as with the Ugandan study.

A study entitled ‘forecasting drug utilization and expenditure in a metropolitan region’, which focussed on the Stockholm, Sweden region aimed to present the forecasting model and predicted growth for all therapeutic agents. Adjustments were made to historical sales data patterns using factors with the potential to increase or decrease expenditure, such as patient deaths, new drugs, prescribing practices or guidelines and structural changes in healthcare. The results were able to predict the percentage of increase of total pharmaceutical expenditure and which classes would have the largest increase [13]. The model used in this research was quite complex and requires robust data, but can be used as a guide for further research in SA.

Other international studies included how pharmaceutical expenditure was managed. In New Zealand, they manage public pharmaceutical expenditure through the pharmaceutical management agency (PHARMAC) which selects drugs that are subsidized by the government. PHARMAC excludes hospital pharmaceuticals. This differs from SA as our government funds healthcare facility pharmaceuticals. The PHARMAC has a Pharmaceutical Schedule which SA calls the EML, these items are subsidized [14]. New Zealand budgeted based on this list, and per this research so did SA. The New Zealand, study unlike this study, did not specifically explore how the budget was translated into pharmaceutical expenditure for the provision of their chosen list.

Another study, ‘the drug budget silo mentality: the Dutch case’, was also based on the budgeting of pharmaceuticals. This study included that the budget was based on the size of the population it served which was in line with the results of this research. The rest of the article focussed on prescription control, price regulation and reimbursement criteria more so then how the budget was allocated to pharmaceuticals [15].

### A comparison of SA budgeting to the WHO budgeting guide

When compared to the WHO budgeting for health guide, SA’s budgeting system was in line with the WHO with a few exceptions. The few areas that were lacking, participants were aware of and making attempts to implement. SA has experienced challenges which prevent some of the WHO recommendations to be met.

Various tasks towards budgeting and expenditure are not mandated and done only on the availability of personnel and resources, are time-constrained and are only done if members take the initiative. Additional members should be allocated to facilitate and assist with these tasks, such as reviewing the demand plan against the actual demand, which is a suggestion in the budget cycle provided by WHO. Challenges still exist with attaining accurate data, systems need to be more robust, so that accurate data can be obtained and important decisions can be made based on correct data.

### What this study adds and recommendations for future studies

The results of this study could be used to guide the budget allocation process and have important applications involving the allocation of the pharmaceutical budget. This research provided valuable information on how the healthcare budget is translated into pharmaceutical expenditure by filling the necessary gaps in knowledge. It has highlighted the deficiencies and challenges in the budgeting process. A process of system improvements through a series of recommendations can commence, which would lead to quality improvements and efficiencies in health systems. This study can help ensure that the healthcare budget is developed with consideration to all aspects, to not disadvantage or under-cater to the needs of the population of SA.

Further research on this topic could include interviews with members at NDoH and National and Provincial Department of Finance to fill the gaps in knowledge. Research on the budget impact on pharmaceutical contract prices, changes and updates, could indicate the influence of contracts on the pharmaceutical budget. This research can serve as a platform for further research on budget formulation and its translation into pharmaceutical expenditure in SA and track improvements and trends over the years. The results can assist other low to middle-income countries in their budget formulation and decision-making processes.

This information needs to be disseminated to health care professionals and the public so that they can work together to follow guidelines that assist with keeping to the budget. Statistics and data collected by health facilities that are used for calculating the budget can be given more focus and performed accurately. Education on guidelines that assist the budget can help with lowering costs and allow for unused allocated funds to be driven into neglected areas.

### Strengths and limitations

This research represented the views of only seven of the nine provinces. Challenges included contacting participants and securing a schedule for interviews due to their very demanding full-time roles. This research included the views of pharmaceutical officials, interviews with finance and budget officials as well as members from all tiers of the healthcare system could paint a more holistic picture of the entire budgeting process.

## Conclusions

This study depicted the knowledge and participation of pharmaceutical services in the budget process. Multiple factors affect the budget and expenditure, which requires constant analysis, monitoring and feedback. Determination of the budget is done by the department of finance; however, pharmaceutical services have an important role in informing the budget and making budgetary negotiations. The principal guiding tool for the provision of medicine is the STG and EML. The PTC plays a vital role in ensuring the healthcare budget is translated into pharmaceutical expenditure for provision on the STG and EML and non-EML items. This study has important applications in guiding the budget process, identifying and resolving challenges and serves as a platform for further research on pharmaceutical budgeting.

## Data Availability

Data upon which conculsions are based in this study are included in this published article in tables, figures and verbatim quotes.

## Abbreviations

AIDS: Acquired immunodeficiency syndrome
ARV: Antiretroviral
ATC: Anatomical Therapeutic Chemical
CEO: Chief Executive Officer
CFO: Chief Financial Officer
CPI: Consumer Price Index
DORA: Division of Revenue Act
EDL: Essential drug list
EML: Essential medicines list
HIV: Human immunodeficiency virus
HOD: Head of Department
MUR: Medicine utilization review
NCD: Non-communicable diseases
NDoH: National Department of Health
NDP: National Drug Policy
NEMLC: National Essential Medicines List Committee
NGO: Non-governmental Organization
NTSG: National Tertiary Services Grant
PFMA: Public Financial Management Act
PHARMAC: Pharmaceutical Management Agency
PHC: Primary Healthcare
PPTC: Provincial Pharmaceutical Therapeutics Committee
PTC: Pharmaceutical Therapeutics Committee
SA: South Africa
SARS: South African Revenue Service
STG: Standard treatment guideline
TB: Tuberculosis
UHC: Universal Health Coverage
WHO: World Health Organization
